# Supplementary material for: Identification of photoperiod-induced specific miRNAs in the adrenal glands of Sunite sheep (Ovis aries)
Source: Front Vet Sci. 2022 Jul 22;9:888207. doi: 10.3389/fvets.2022.888207 (PMC9354845; doi:10.3389/fvets.2022.888207)
Supplement: Supplementary file 1 [file Data_Sheet_1.ZIP › Supplementary file/miRDeep2 file.zip!/miRDeep2 file/Know_Structure/LP42A2/oar-let-7a.pdf]

miRBase precursor : oar-let-7a
Total read count : 318121
oar-let-7a read count : 316936
remaining reads : 1185

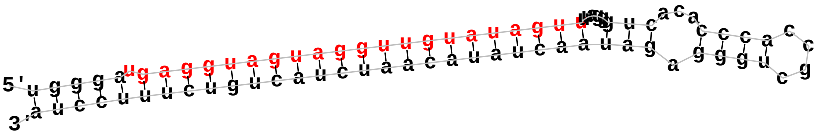

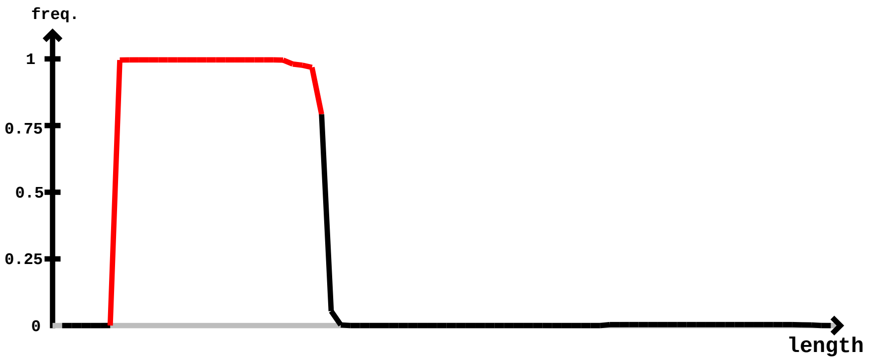

oar-let-7a

5'- ugggaugagguaguagguuguauaguuuuagggucacacccaccgcugggagauaacuauacaaucuacugucuuuccua -3'	exp		
(((((.(((((((((((((((((((((.....(((...((((....)))).)))))))))))))))))))))))))))))	reads	mm	sample
....augagguaguagguugua..	1	0	seq
....augagguaguagguuguau...	1	0	seq
....augaAguaguagguuguau...	1	1	seq
....Uugagguaguagguuguaua..	1	1	seq
....augagguaguagguuguauag...	1	0	seq
....augagguaguagguuguauagu..	11	0	seq
....Uugagguaguagguuguauagu..	6	1	seq
....Uugagguaguagguuguauaguu...	10	1	seq
....augagguaguagguuguauaguu...	21	0	seq
....augagguaguagguuguauaguuu..	2	0	seq
....Uugagguaguagguuguauaguuu..	1	1	seq
.....ugagguUguagguugua..	1	1	seq
.....uUagguaguagguugua..	1	1	seq
.....ugagguaguagguugua..	239	0	seq
.....ugagguaguaAguugua..	6	1	seq
.....ugaggCaguagguugua..	1	1	seq
.....ugagguagGagguugua..	28	1	seq
.....ugaAguaguagguugua..	1	1	seq
.....ugagguaguUgguugua..	1	1	seq
.....ugagguaguaAguuguau...	18	1	seq
.....ugagguUguagguuguau...	1	1	seq
.....ugagguagGagguuguau...	410	1	seq
.....ugagguaguagguuUuau...	7	1	seq
.....ugaAguaguagguuguau...	31	1	seq
.....ugagguaguagguuguau...	4249	0	seq
.....ugagguaguagguuguaua..	1204	0	seq
.....ugagguagGagguuguaua..	169	1	seq
.....ugagguagGagguuguauag...	383	1	seq
.....ugagguaguagguuguauag...	2189	0	seq
.....ugagguaguagguuguauagu..	48831	0	seq
.....ugagguagGagguuguauagu..	7059	1	seq
.....ugagguaguagguuguauaguu...	213024	0	seq
.....ugagguagGagguuguauaguu...	21638	1	seq
.....ugagguaguagguuguauaguuu..	16508	0	seq
.....ugagguaguagguuguauaguuuA...	131	1	seq

oar-let-7a

ugggaugagguaguagguuguauaguuuuagggucacacccaccgcugggagauaacuauacaaucuacugucuuuccua			
.....ugagguaguagguuguauaguuuu...	561	0	seq
.....ugagguaguagguuguauaguuuua..	2	0	seq
.....ugagguaguagguuguauaguuuuaggg...	2	0	seq
.....ugagguaguagguuguauaguuuuagggu..	1	0	seq
......gagguaguagguuguau...	4	0	seq
......gagguaguagguuguaua..	2	0	seq
......gagguaguagguuguauag...	1	0	seq
......gagguaguagguuguauagu..	26	0	seq
......gagguagGagguuguauagu..	7	1	seq
......gagguaguagguuguauaguu...	113	0	seq
......gagguagGagguuguauaguu...	14	1	seq
......gagguaguagguuguauaguuu..	8	0	seq
.......agguaguagguuguauaguu...	3	0	seq
.......aggCaguagguuguauaguu...	1	1	seq
........gguaguagguuguauagu..	1	0	seq
........gguaguagguuguauaguu...	3	0	seq
.........guaguagguuguauaguu...	1	0	seq
..........uaguagguuguauaguu...	2	0	seq
..........uaguagguuguauaguuuua..	1	0	seq
...........................uuagggucacacccaccgcugg...............................	2	0	seq
...........................uuagggucacacccaccgcuggg..............................	5	0	seq
...........................uuagggucacacccaccgcuggga.............................	10	0	seq
...........................uuagggucacacccaccgcugggaga...........................	1	0	seq
....auaacuauacaaucuacugucu......	2	0	seq
....auaacuauacaaucuacugucuuu....	1	0	seq
....aacuauacaaucuacugucuuu....	1	0	seq
....cuauacaaucuacugucu......	4	0	seq
....cuauacaaucuacugucuu.....	14	0	seq
....cuauacaaucuacugucuuu....	286	0	seq
....cuauacaaucuacuguUuuuc...	1	1	seq
....cuauacaaucuacugucuuuc...	162	0	seq
....cuauacaaucuacugucuuucc..	631	0	seq
....cuauacaaucuacugucuuuccu.	30	0	seq
....uauacaaucuacugucuuA....	1	1	seq
....uauacaaucuacugucuuuc...	1	0	seq
....uauacaaucuacugucuuucc..	4	0	seq
....uauacaaucuacugucuuuccu.	17	0	seq
....auacaaucuacugucuuucc..	2	0	seq
....auacaaucuacugucuuuccu.	6	0	seq
....auacaaucuacugucuuuccua	1	0	seq
